# Targeting Highly Structured RNA by Cooperative Action of siRNAs and Helper Antisense Oligomers in Living Cells

**DOI:** 10.1371/journal.pone.0136395

**Published:** 2015-08-26

**Authors:** Mariola Dutkiewicz, Agata Ojdowska, Jakub Kuczynski, Vanessa Lindig, Heinz Zeichhardt, Jens Kurreck, Jerzy Ciesiołka

**Affiliations:** 1 Department of RNA Biochemistry, Institute of Bioorganic Chemistry, Polish Academy of Sciences, Poznan, Poland; 2 Institute of Virology, Campus Benjamin Franklin, Charite´—University Medicine, Berlin, Germany; 3 Institute of Biotechnology, Department of Applied Biochemistry, Berlin University of Technology, Berlin, Germany; Wuhan University, CHINA

## Abstract

RNA target accessibility is one of the most important factors limiting the efficiency of RNA interference-mediated RNA degradation. However, targeting RNA viruses in their poorly accessible, highly structured regions can be advantageous because these regions are often conserved in sequence and thus less prone to viral escape. We developed an experimental strategy to attack highly structured RNA by means of pairs of specifically designed small interfering RNAs and helper antisense oligonucleotides using the 5’ untranslated region (5’UTR) of coxsackievirus B3 as a model target. In the first step, sites accessible to hybridization of complementary oligonucleotides were identified using two mapping methods with random libraries of short DNA oligomers. Subsequently, the accessibility of the mapped regions for hybridization of longer DNA 16-mers was confirmed by an RNase H assay. Using criteria for the design of efficient small interfering RNAs (siRNA) and a secondary structure model of the viral 5’UTR, several DNA 19-mers were designed against partly double-stranded RNA regions. Target sites for DNA 19-mers were located opposite the sites which had been confirmed as accessible for hybridization. Three pairs of DNA 19-mers and the helper 2’-O-methyl-16-mers were able to effectively induce RNase H cleavage *in vitro*. For cellular assays, the DNA 19-mers were replaced by siRNAs, and the corresponding three pairs of siRNA-helper oligomer tools were found to target 5’UTR efficiently in a reporter construct in HeLa cells. Addition of the helper oligomer improved silencing capacity of the respective siRNA. We assume that the described procedure will generally be useful for designing of nucleic acid-based tools to silence highly structured RNA targets.

## Introduction

In the last decade, RNA interference (RNAi) has emerged as a new technique that is in the process of developing into a new class of therapeutics [1–3]. Extensive research on the mechanism of RNAi and the application of this approach to gene silencing have led to the establishment of rules for the design of active siRNAs. It has been shown that RNA target accessibility is a very important factor influencing the efficiency of RNAi-mediated RNA degradation [4–8]. Tightly associated RNA structures can severely limit or even preclude the targeting of small interfering RNAs (siRNAs), even if their sequences have been carefully chosen according to the best known thermodynamic criteria. This may also at least partially explain why up to 6% of targets in the human genome and many structured viral RNAs are refractory to silencing by siRNAs [9].

In dynamically changing viral genomes the best regions to target are highly conserved non-coding RNA stretches, since targeting such regions makes viral escape less likely. However, non-coding RNA regions are frequently involved in the formation of highly structured RNA elements that enables their regulatory function and, at the same time, makes it difficult for oligonucleotide-based tools to access them.

Probing the accessibility of tightly structured RNA regions for oligomer hybridization can be helpful in siRNA design. In some cases, however, a nucleotide sequence fulfilling thermodynamic criteria for active siRNAs might be impossible to identify in accessible regions. It may then be unavoidable to target partly double-stranded, less accessible regions of the target RNA. The application of modified siRNAs, i.e. LNA-modified siRNAs (*locked nucleic acids*), which exhibit a high affinity to the RNA target, has been shown to improve silencing efficiency [10]. Another way to overcome tight RNA structures is with the use of a helper antisense oligomer, “a clamp”, that is able to bind the RNA target with high affinity, thereby opening its double-stranded stem and making the target site more accessible for hybridization of the siRNA. This method has been shown to work well in cell lysates with the use of 2’-O-methyl-20-mer [11].

An example of a highly structured, difficult to target RNA is the 5’ untranslated region (5’UTR) of coxsackievirus B3 (CV-B3). CV-B3 is a heart pathogen against which no specific treatment has been approved to date. Characteristic for a member of the genus *Enterovirus* of the *Picornaviridae* family, CV-B3 contains a single stranded RNA genome in the plus-strand orientation. It encodes a single open reading frame flanked by untranslated regions [12–13]. The 5’UTR contains an internal ribosome entry site (IRES) that directs the translation process and a cloverleaf structure that regulates viral replication [14]. A number of siRNAs have been tested against the 5’UTR of CV-B3 [10, 15–18], but only one of them exerted high antiviral activity [10].

Here, we describe a rational design procedure for obtaining active siRNAs targeting highly structured RNA regions. We investigated whether binding of a helper oligomer to the accessible side of a double-stranded stem increases the efficiency of siRNA to binding to the opposite strand of the stem. The 5’UTR of CV-B3 served as a model target in these studies. For cellular assays, this region of the viral RNA was fused with the mRNA encoding humanized *Renilla reniformis* green fluorescent protein (hrGFP). We show that the silencing efficiency of siRNA can be improved substantially by the simultaneous application of 2’-O-methyl-modified helper oligomers. The procedure of designing cooperating combinations of oligonucleotide tools could be generally helpful for targeting highly structured RNAs.

## Materials and Methods

The materials used in this study were obtained from the following sources: (γ-^32^P)ATP (4600 Ci/mmol) from Hartmann Analytic (Braunschweig, Germany) and all the chemicals were purchased from Sigma-Aldrich (St. Louis, Missouri, USA), Serva Electrophoresis (Heidelberg, Germany) or BioShop (Burlington, Canada). All enzymes were purchased from Fermentas (ThermoFisher Scientific, Waltham, Massachusetts, USA) unless stated otherwise. Unmodified DNA oligomers were purchased from Oligo Service IBB PAS (Warsaw, Poland) and they were deprotected after synthesis and purified on 8% polyacrylamide gels.

### dsDNA templates and synthesis of 5′UTRcvb3 RNA

In order to obtain the dsDNA template (nucleotides 1–742 of the CV-B3 Nancy strain under control of the T7 promoter), single-stranded DNA complementary to the 5′ portion of CV-B3 genome was synthesized by reverse transcription as described previously [19]. Subsequently, cDNA was amplified by PCR involving specific primers (T7fcvb: 5'-TAATACGACT CACTATAGGT TAAAACAGCC TGTGGGTTG-3’; Rcvb: 5'-TTTGCTGTAT TCAACTTAAC AATG-3’). As a result, dsDNA encoding the desired RNA sequence was generated, containing the T7 promoter at the 5’end: dsDNA_5′UTRcvb3. The reverse transcription and PCR reactions were performed according to standard protocols. The reaction products were purified by phenol/chloroform (1:1) extraction, precipitated with ethanol, and the obtained dsDNA template was dissolved in TE buffer.

Transcription reactions were performed as described previously [20]. The synthesized 5′UTRcvb3 RNA was checked for size, integrity and homogeneity on denaturing agarose gel, and purified with RNeasy MinElute Cleanup Kit columns (Qiagen, Hilden, Germany). If necessary, it was labeled with ^32^P at its 5′ end with polynucleotide kinase according to standard procedures.

### Mapping of extendible sites by means of reverse transcription with random oligonucleotide libraries (RT-ROL)

According to a protocol described in [21] the renatured RNA of 5’UTRcvb3 was separately subjected to hybridization with two short-DNA-oligomer libraries, one of them containing oligonucleotides with 8 randomized positions, while the other contained 12 randomized nucleotide positions. Both libraries contained a tag-sequence at the 5’-end of each oligomer. Additional reactions were performed with an anti-tag oligomer, i.e. an oligomer that blocks the complementary sequence at the 5’ end of oligonucleotides during the DNA-RNA hybridization. Those oligomers which were able to bind to the accessible sites in 5’UTRcvb3 were then extended by RevertAid reverse transcriptase. The generated cDNA fragments were subsequently amplified by PCR with pairs of primers, one of which was the tag primer and the other was the 5’-^32^P-labeled RNA-specific primer (S1 Table). RT-ROL products were analyzed by sequencing gel electrophoresis and run along sequencing lines as described in [20].

Estimation of the extendible (accessible to hybridization) sites was performed on the basis of RNA sequencing and the length and location of a random sequence in each oligomer library.

### Mapping of RNA accessibility to hybridization with DNA 6-mers libraries and RNase H digestion

Mapping of RNA accessibility to hybridization with DNA 6-mers libraries and RNase H digestion was performed as described previously [22–24]. Briefly, prior to digestion with *E*. *coli* RNase H, the 5’-^32^P-labeled RNA was renatured in an appropriate buffer by heating at 65°C for 5 min and slowly cooling to 37°C. Subsequently, RNase H was added and the cleavage reactions were initiated by adding the DNA 6-mer libraries to separate RNA samples. The mixtures were incubated at 37°C for 10 or 30 min. The reactions were quenched with equal volumes of 20 mM EDTA/7 M urea mixture and frozen on dry ice. The digestion products were analyzed by electrophoresis on 12% polyacrylamide, 8.3 M urea gels. Autoradiography was performed using phosphoimager (Fujifilm, Tokyo, Japan). Products of RNase H digestion reactions were run on polyacrylamide gels along with the products of alkaline RNA hydrolysis and limited T1 ribonuclease digestion of the same RNA according to protocol described previously [23–24].

### siRNA target identification

The 5’UTR sequence of CV-B3 ranging from nucleotide 1 to 742 (according to GenBank accession No. JX312064.1) was searched for 19-nucleotide-long stretches according to the following principles based on the Reynolds criteria [25]: A at position 3, U at position 10, a “non G” at position 13, A or U at position 19, several A’s or U’s at positions 15–18, a GC content between 30 and 52%. Less strict rules were used to find more potential target sites: (a “non G” at position 13, A or U at position 19, several A’s or U’s at positions 15–18, GC content between 30 and 52%). The appropriate thermodynamic asymmetry was checked, which means that the 5’ terminal end of the guide strand in the prospective siRNA duplex should exhibit lower free energy than the 3’ terminal end.

### Antisense DNA molecules and RNase H assay

The RNase H assay was performed as described previously [10, 26]. The *in vitro* transcribed RNA of 5’UTRcvb3 was refolded and hybridized with a five-fold excess of antisense DNA oligonucleotides. Duplexes were incubated in the presence of E.coli RNase H (Promega, Madison, USA) for 15 min at 37°C. Reactions were quenched with EDTA, mixed with loading dye and run in agarose gel electrophoresis. In case of mixtures composed of two different oligomers, each oligomer was applied at a final concentration of 5 μM.

### Thermodynamic calculations of DNA-RNA and RNA-RNA duplexes

The thermodynamic properties of oligomer binding to the RNA target were analyzed by *RNAstructure 5*.*4* computer program [27]. An *OligoWalk* application was used to calculate the free energy values of the created DNA-RNA or RNA-RNA duplexes. The binding energies of three DNA 19-mers and three RNA 16-mers with 5’UTRcvb3 RNA sequence were analyzed: DNA_162, DNA_213, DNA_420, as well as and RNA5, RNA8, and RNA9 (their sequences corresponded to 2’-O-methyl-16-mers: *me_5*, *me_8*, and *me_9*, respectively). A “break local structure” mode was used.

### Cell culture

For the reporter assay experiment, HeLa cells (human cervical carcinoma) were grown in monolayers in an RPMI medium containing 10% heat-inactivated fetal calf serum (FBS), 100 u/ml penicillin and 100 μg/ml streptomycin and with 250 ng amphotericin B per mL (Sigma-Aldrich). Cells were cultivated at 37°C in a humidified atmosphere with 5% CO_2_. Cells were passaged 24 hours before transfection.

For experiments involving inoculation with the CV-B3, HeLa cells were grown as described elsewhere [17].

### DMS probing *in vivo*

A permissive HeLa cell line (courtesy of Dr. R. R. Rueckert, Madison, Wis.) was inoculated with the Nancy strain of CV-B3 (ATCC VR-30) at a multiplicity of infection (moi) of 1. A virus titer of 1.3 x 10^8^ PFU/ml was used. 5 hours post-infection, cells were harvested, suspended in PBS and divided into three samples. The *in vivo* DMS treatment was performed on the basis of protocols published elsewhere [28–31]. Briefly, DMS at a final concentration of 0.25% was added to two samples, prior to or after treatment with 0.7 M ß-mercaptoethanol. The third, control sample was only treated with 0.7 M ß-mercaptoethanol. After 10 min incubation at 37°C, the samples were centrifuged, and the cell pellet was washed several times with 0.7 M ß-mercaptoethanol. The cell pellet was immediately subjected to RNA extraction using the TRI-reagent (Sigma-Aldrich). After purification, the isolated total RNA was resuspended in RNase-free water.

To determine sites in viral RNA that were modified by DMS treatment, reverse transcription reactions were performed with 7 μg of the total RNA and virus-specific 5′-end-^32^P-labeled DNA primers (S3 Table). Dideoxy sequencing markers were generated with *in vitro* transcribed RNA from the dsDNA_5′UTRcvb3 template described above. The procedure was described in [20] with the only difference being the use of RevertAid M-MuLV reverse transcriptase and FLA 5100 image analyzer (Fujifilm, Tokyo, Japan).

### GFP fusion constructs

The fusion construct of hrGFP gene and the 5’UTR, a viral subgenomic fragment of CV-B3 ranging from nucleotide 1 to 742, was generated by standard cloning into phr-GFP-1 vector (Stratagene, La Jolla, CA) according to the manufacturer’s instructions in multiple cloning site 2. The insert representing the 5’UTR sequence was synthesized by extension of the dsDNA_5′UTRcvb3 (described above) at both ends with restriction sites EcoRI and KpnI in a standard PCR reaction with specific primers (ER1T7cvb: 5'-ACAGAATTCT AATACGACTC ACTATAGGTT AAAACAGCCT GTGGGTTG-3', KpnIRcvb: 5'- GATGGTACCT TTGCTGTATT CAACTTAAC AATG-3’).

### siRNAs, helper antisense oligomers and transfection

The specific siRNA duplexes containing symmetrical 3’-dTdT overhangs and with ON-TARGET modifications (Table 1) as well as the control siRNA1 ON-TARGET were purchased from DHARMACON (Thermo Fisher Scientific). The specific 2’-O-methyl-16-mers and the negative control 2’-O-methyl-oligomer were purchased from FUTUREsynthesis (Poznan, Poland). Negative control oligomers have no matches either in the viral or the human genome. For transfection, HeLa cells were plated in 24-well plates at a density of 2 x 10^5^ cells/well in a volume of 500 ml without antibiotics. At the same time (reverse transfection protocol), cells were co-transfected with the appropriate amount of siRNAs (0, 0.1 or 0.25 nM), 2’-O-methyl-16-mers (0 or 40 nM), and 0.5 μg of hrGFP-5’UTRcvb3 fusion construct. An amount of 1.75 μl of Lipofectamine 2000 (Thermo Fisher Scientific) was applied per well, following the manufacturer’s instructions. Following transfection, cells were incubated at 37°C in humidified atmosphere for 24 hours.

**Table 1:** Helper antisense oligonucleotides and siRNAs against the 5’UTRcvb3 RNA.

**Helper antisense oligonucleotide**	**sequence 5’-3’ (all residues 2’-O-methyl)**
*me_ 8*	UUG AUA CUC AGU CCG G
*me_ 9*	AGU AGU UGG CCG GAU A
*me_ 5*	CUG ACU GUU GAU CGG U
*me_ C*	ACA CGU UCG GAG AAU U
**siRNA:**	**sense strand sequence 5’-3’**
siRNA_420	CGA AGA GUC UAU UGA GCU A TT
siRNA_213	GGU UGA AGG AGA AAG CGU U TT
siRNA_162	UGA UCA AGC ACU UCU GUU A TT
siRNA_C	UUC UCC GAA CGU GUC ACG U TT

### Western blotting

24 hours after transfection, cells were lysed in 24-well plates with laemmli sample buffer. After boiling the lysate at 95°C for 5 min, proteins were separated on a 15% (w/v) polyacrylamide gel. Transfer of proteins to PVDF membranes (Pierce, Rockford, IL) was performed with a semi-dry blotter (Bio-Rad, Hercules, California, U.S.A.). Subsequently, membranes were incubated with a monoclonal rabbit hrGFP antiserum (1:5000; Stratagene) overnight in TTBS buffer at 4°C. Secondary antibodies conjugated with alkaline phosphatase (Pierce) were used at a dilution of 1:5000 in 5% skim milk. Detection by chemoluminescence was achieved using ECL Kit (Pierce). To confirm equal loading of samples, membranes were re-probed with a monoclonal mouse antibody specific for GAPDH (Santa Cruz Biotechnology). At least three independent experiments were performed. For the experiment with 0.25 nM concentration of siRNAs the band-density measurements were performed. The values obtained for hrGFP were then normalized to the values obtained for GAPDH in each sample line. The p-values were calculated using Student’s t-test.

### Fluorescence intensity reading

HeLa cells were co-transfected using the identical protocol as described in the previous section with the only difference being that the cells and transfection mixture were divided into four wells of 96-wells plate. Black plates with transparent bottom were used (Perkin Elmer, Waltham, MA). At 24 h after transfection, cells were washed with PBS and subjected to fluorescence intensity reading of the expressed hrGFP in a plate reader (EnSpire, Perkin Elmer). The excitation wave length as well as the emission wave length were established experimentally in the plate reader and set at 492 nm and 507 nm, respectively. The fluorescence intensity measurement was performed from the bottom side of the plate. The scanning-mode was applied, which means that 80 different points of each well were measured and the average values of the fluorescence intensity were then calculated for each well. At least three independent experiments were performed, each in duplicate. The p-values were calculated using Student’s t-test.

## Results

### Mapping of accessible sites for oligonucleotide hybridization using short oligonucleotide libraries

**Fig 1:**
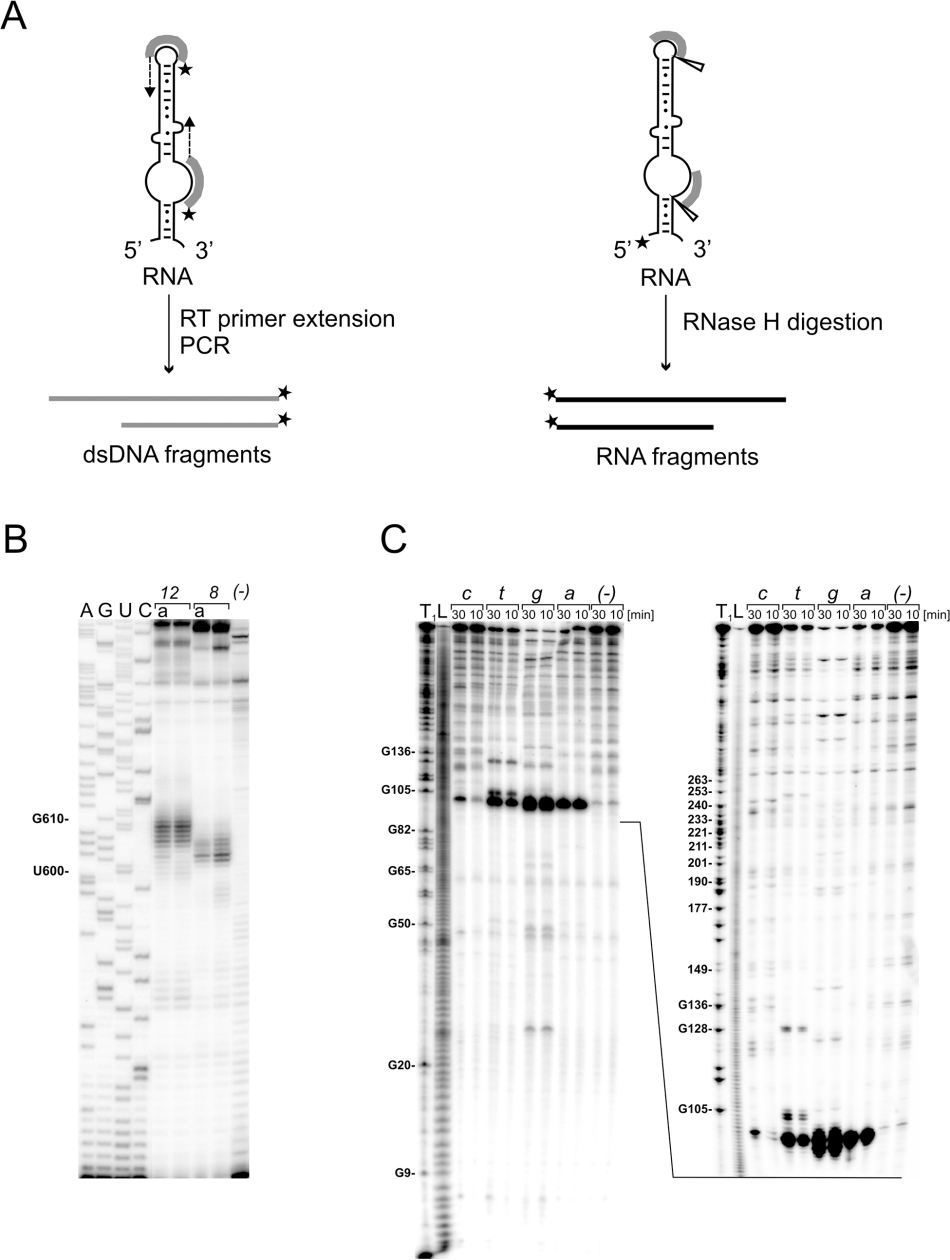
Mapping of accessible sites for oligonucleotide hybridization in the 5’UTRcvb3 RNA. (A) Schematic representation of the two methods applied to map regions which are accessible to oligonucleotide hybridization. Thick grey lines—complementary oligomers hybridized to RNA; star—radioactive label; dashed arrow—a direction of primer extension; triangle—a site of RNase H induced RNA cleavage. Grey or black lines with an arrow at the end—dsDNA or RNA products of the procedures, respectively. (B) Analysis of Reverse Transcription with Random Oligonucleotide Libraries (RT-ROL) products by sequencing gel electrophoresis. The RT-ROL products were generated with 8- and 12-mer libraries followed by PCR amplification with the radiolabeled RNA-specific primers and the tag primer. Lanes: *(-)*—reaction control without DNA library; A, G, U, C—RNA sequencing lines; *8*—random 8-mer library; *12*—random 12-mer library; a—reactions in the presence of the antitag-oligomer. Selected nucleotide residues are labeled on the left. Figure shows a typical autoradiogram. The other autoradiograms are shown in S1 Fig. (C) Cleavage sites induced by RNase H in the presence of semi-random libraries of deoxynucleotide 6-mers: *a*, *c*, *t*, or *g*. The reactions were carried out at 37°C for 10 and 30 min with the 5'-end-^32^P-labeled 5’UTRcvb3 RNA: Lanes: *(-)*—reaction control without DNA library; L—formamide ladder; T_1_—limited hydrolysis by RNase T1. Selected guanine residues are labeled on the left. The short and long run of the gel is shown.

**Fig 2:**
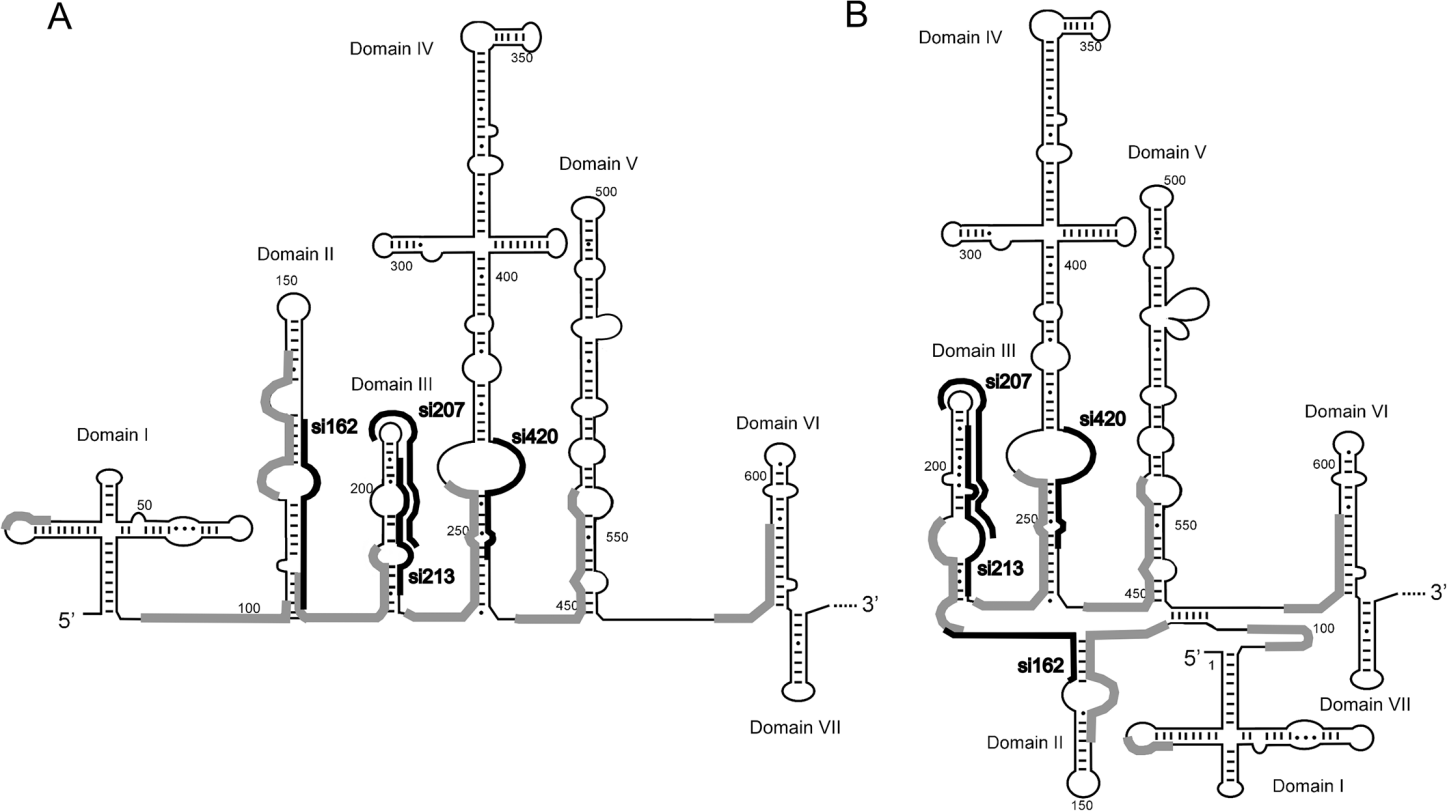
Sites in the 5’UTRcvb3 RNA that are accessible for hybridization to complementary oligonucleotides. Thick grey lines indicate sites that were mapped as accessible in the secondary structure models proposed by Bailey and Tapprich (2007). Potential target sites for siRNAs are indicated by thin black lines along the structure. (A) The structure model of the 5' UTR of CV-B3 generated by comparative sequence analysis and energy minimization. (B) Experimentally validated structure model.

Prior to applying the oligonucleotide-based strategy to target the highly structured 5’UTR of CV-B3 in living cells, we mapped sites accessible to hybridization of complementary oligonucleotides in the model RNA fragment, 5’UTRcvb3. This step was supposed to be helpful for both siRNA and helper oligomer design. The RNA was obtained by *in vitro* transcription using a DNA template encoding the sequence of 5’UTRcvb3 corresponding to the 5’UTR region of the CV-B3 virus, Nancy strain. We applied two methods in order to identify the sites accessible to hybridization within the 5’UTR (Fig 1A). One method used the two semi-random libraries of DNA oligomers with 8 and 12 randomized nucleotide positions, respectively, and a reverse transcriptase enzyme that extended only those oligomers which were able to hybridize to the RNA target. The obtained products were then amplified in a PCR reaction, in which one of the primers was radioactively labeled, separated by gel electrophoresis and identified on the gel (Fig 1B). The other method used the four semi-random libraries of DNA 6-mers and RNase H that cleaved the 5’-radiolabeled RNA within RNA-DNA heteroduplexes (Fig 1C). The latter approach was applied to elucidate the accessibility of the 5’ terminus of the 5’UTR, since this region escaped characterization with the primer-extension method. The sites accessible to hybridization of complementary oligonucleotides within the 5’UTRcvb3 RNA were displayed in the two secondary structure models of the viral 5’UTR which have been proposed in the literature (Fig 2). The model of Bailey and Tapprich [32] assumes the presence of long-range interactions between distant domains (Fig 2B). It is remarkable that a majority of the domains I, IV, V, VI, as well as VII turned out to be poorly accessible to oligomer hybridization in our assays. The single-stranded linkers between domains appeared to be accessible, with the exception of linkers between domains V-VI and II-III (according to Fig 2B) which were accessible only to a small extent. Surprisingly, accessible were also partly double-stranded regions located at the 5’ sides of the domains II, III, IV, V and VI. The region with the highest accessibility for complementary oligonucleotides was the located in between the cloverleaf structure (domain I) and the rest of the RNA molecule. Strong, multiple bands close to nucleotide in position 100 were observed on the gel (Fig 1C).

### Searching for appropriate target sites for the siRNA approach

The sequence of the 5’UTR of CV-B3 was searched for the best potential target sites for siRNA targeting. Several criteria specified earlier were taken into account in this selection. Remarkably, only nine 19-nucleotide-long stretches within the 742-nucleotide-long RNA region reached the score of “6” or more according to the Reynolds criteria [25, 33]. Applying more flexible rules, 87 target sites were identified. However, when the appropriate thermodynamic asymmetry and lack of nucleotide tracks (3 x C/G or more; or 4 A/T or more) were taken into account, only 24 sites remained. Those which have already been tested previously or the sites which encompassed the positions of frequent point mutations published in the GeneBank were also disregarded. Finally we ended up with 19 novel potential target sites for the siRNA approach. Remarkably, most of these sites were located outside the regions accessible to hybridization or only partially overlapped with them.

Four of the potential siRNA-sites were displayed in Fig 2, which shows how they correspond to the sites mapped as accessible to oligonucleotide hybridization. In domain II, the siRNA target sites starting at position 162 (si162) was located on the opposite side of the stem that was shown to be accessible between nucleotide positions 119 and 143. In domain III, the siRNA target sites starting at positions 207 and 213 (si207 and si213) were located on the side opposite to the accessible region between nucleotides 177 and 194, and in domain IV, the siRNA target sites beginning at positions 413, 416 and 420 (only si420 is shown in the figure) were located on the side opposite to the region spanning nucleotides 235 and 257, which was accessible to hybridization (Fig 2).

### Binding of potential helper antisense oligomers analyzed by RNase H digestion

We noticed that several potential siRNA target sites were located in the double-stranded stem segments opposite to the sites accessible to oligonucleotide hybridization. This observation suggested an opportunity of using helper antisense oligomers to make the opposite sides of the stems more accessible. Binding of a helper oligomer might destabilize the stem, allowing RNA-induced silencing complex (RISC) loaded with the siRNA to hybridize to the other side of the stem [11].

**Fig 3:**
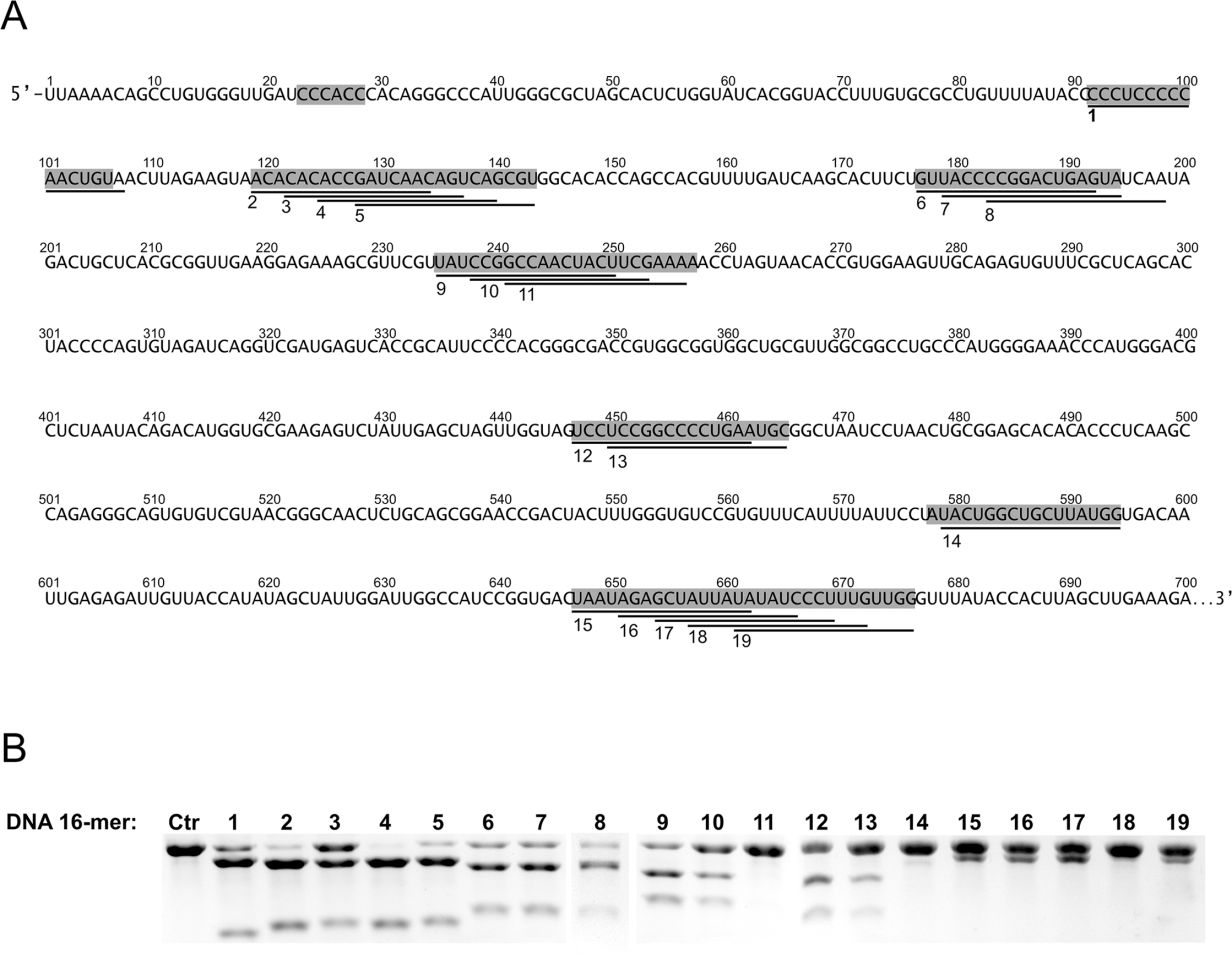
Induction of RNase H cleavage by potential helper oligomers. (A) Nucleotide sequence of the 5’UTRcvb3 RNA. Sites mapped as accessible to oligonucleotide hybridization are indicated with gray rectangles. Target sites of DNA 16-mers, potential helper oligomers, are marked with black lines below the sequence and numbered 1–19. (B) Representative agarose gels showing degradation of 5’UTRcvb3 RNA by RNase H in the presence of one of the DNA 16-mers. Numbers correspond to target sites indicated in the sequence in (A). Ctr—control reaction without DNA oligonucleotide.

To design helper antisense oligomers which would be able to destabilize the double-stranded stems of the targeted RNA, we tested the binding efficiency of several DNA 16-mers complementary to the 5’UTRcvb3 using the RNase H assay. Nineteen DNA 16-mers were chosen for the test based on our previous mapping results and in several cases the oligomers were shifted in the 3’ direction by 2, 3 or 4 nucleotides (Fig 3A). It turned out that several oligonucleotides were capable of inducing efficient RNase H cleavage upon binding: these were oligomers Nos. 1, 2 and 4–9. The upper band on the gel corresponding to the full-length RNA was degraded in those cases (Fig 3B). The 16-mers most efficient at inducing RNase H activity, were oligomers Nos. 2, 4 and 5, complementary to domain II. Unexpectedly, three other oligomers Nos. 11, 14 and 18 were completely inactive in inducing RNA degradation, although they were directed into sites mapped previously as accessible to hybridization. Eight other oligomers Nos. 3, 10, 12, 13, 15–17, and 19, were also inefficient in inducing RNase H activity. These results highlight the need for testing oligomers of the final length for the ability to bind RNA regions mapped as accessible with short-oligomer-libraries. The sequences of all oligomers are shown in S2 Table.

### Opening the structure of targeted RNA by binding the helper oligomers *in vitro*

**Fig 4:**
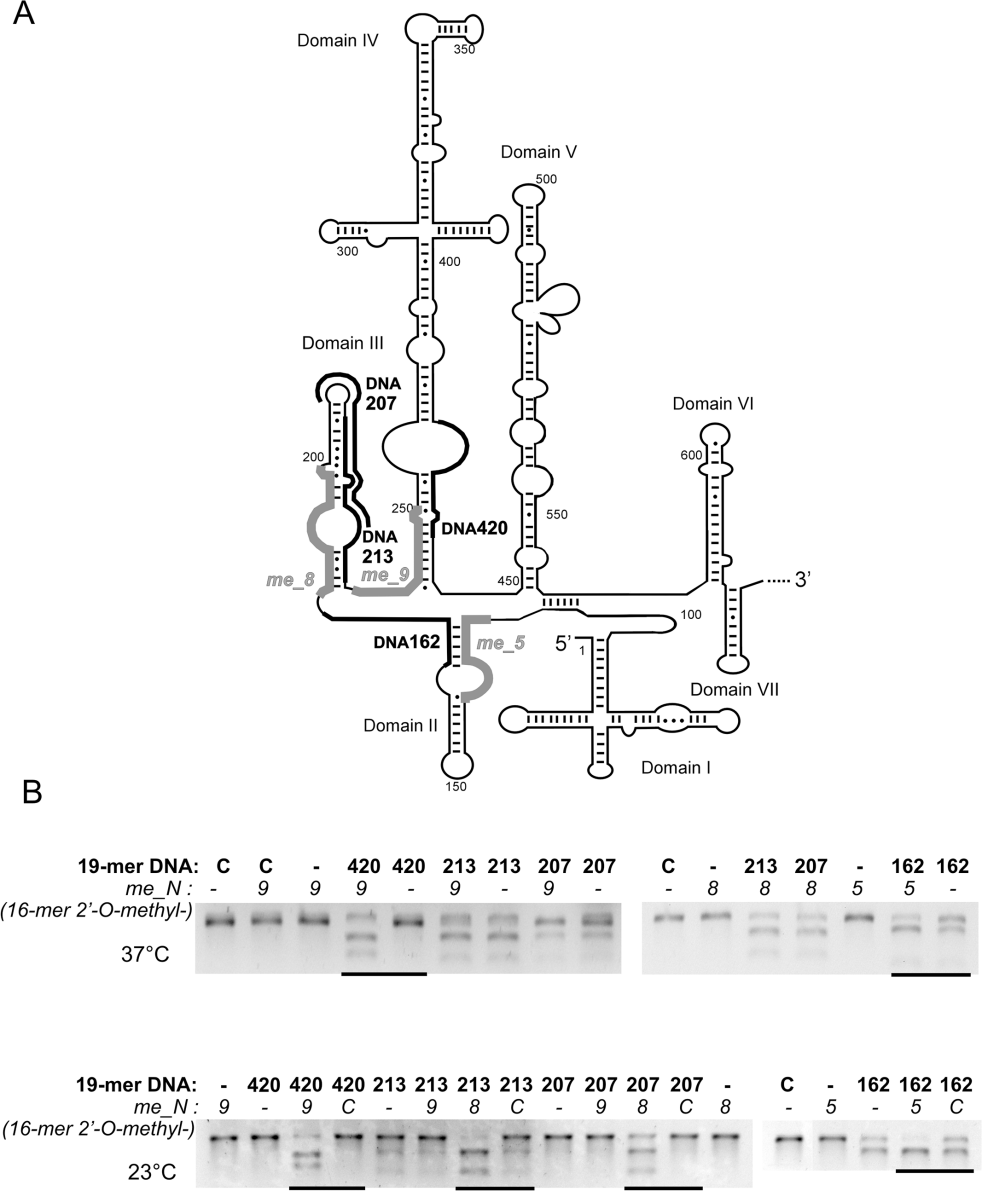
Opening of the new accessible sites for oligonucleotide hybridization in the tightly structured 5’UTRcvb3 *in vitro*. (A) Schematic secondary structure model of the 5'UTR of CV-B3 according to Bailey and Tapprich (2007). Target sites of 2’-O-methyl-16-mers are indicated with grey thick lines and target sites of DNA 19-mers are indicated with black lines. (B) Representative agarose gels showing digestion products of the 5’UTRcvb3 RNA by RNase H in the presence of particular DNA 19-mer. Numbers denote particular oligonucleotides present in the mixture: bold number—name of DNA 19-mer; number in italics—name of a helper oligomer (2’-O-methyl-16-mer). C or *C*—control reaction with an unspecific oligonucleotide. Upper panel—reactions performed at 37°C; lower panel—reactions performed at 23°C.

In order to establish whether binding of the selected 16-mers to the 5’UTRcvb3 RNA could induce opening of the tight structure and render additional sites accessible to oligonucleotide hybridization, the RNase H-assays were again performed, as well as the thermodynamic calculations described below. Binding of helper oligomers to the 5’ sites of double-stranded structural elements of domains II, III and IV was expected to result in improved hybridization of DNA 19-mers on the opposite sides. The length and nucleotide sequences of DNA 19-mers corresponded to guide strands of potential siRNAs. The DNA oligomers were complementary to the RNA target starting at nucleotide positions 420, 213, 207, and 162, respectively (Figs 2 and 4 and S2 Table). To open the structure of domains II, III, and IV, we applied the three 2’-O-methyl-16-mers: *me_5*, *me_8* and *me_9*, respectively, of sequences identical to the previously tested DNA 16-mers Nos. 5, 8 and 9 (Table 1 and S2 Table). Importantly, the 2’-O-methyl chemical modification of oligomers prevents them from inducing RNase H activity at their binding sites and results in stronger RNA binding. The 5’UTRcvb3 RNA was targeted with specific 2’-O-methyl-16-mers in combinations with unmodified DNA 19-mers. Thus, in these experiments, the observed levels of RNA degradation reflected the capability of the DNA 19-mers to bind to the RNA target (Fig 4). Two representative agarose gels shown in Fig 4 illustrate the degradation of 5’UTRcvb3 RNA by RNase H. The reaction was performed at two different temperatures, 37°C and 23°C. In the living cell, possible tertiary interactions within an RNA molecule or other interactions with cellular proteins could potentially stabilize the target RNA structure and make it harder to open by antisense oligomers. The temperature of 37°C corresponded to physiological conditions, whereas at 23°C the structure is stabilized. As negative controls, unspecific 2’-O-methyl-16-mer and an DNA 19-mer were applied separately or in mixtures with other tested oligomers.

In the gels shown in Fig 4B, the upper bands represent the full-length RNA which remained intact in the negative control lines. The DNA 19-mer no. 420 did not induce RNA degradation neither alone nor mixed with the unspecific control oligomer. However, in a mixture with a helper oligomer, *me_9*, a significant level of RNA degradation was observed at both temperatures tested. A similar effect was observed for oligomer no. 207 and its mixture with *me_8*. A slight difference was that at 37°C the oligomer alone was able to induce very weak RNA cleavage as well. That the 19-mers DNA_420 and DNA_207 alone were not able to induce RNA degradation was expected, since they targeted sites mapped as inaccessible to hybridization. Oligomers DNA_162 and DNA_213 were partially effective at 37°C although they also targeted sites mapped as inaccessible. The possible explanation is that being longer than the short oligomers used during accessibility mapping these oligomers were able to hybridize more effectively.

Although oligomer no. 213 was inefficient at 23°C, the mixture of oligomers no. 213 and *me_8* induced significantly stronger RNA degradation at 23°C in comparison with the mixture of oligomers no. 207 and *me_8*. Oligomer no. 162 was moderately effective, but a significant increase of RNA degradation was observed when it was used in combination with *me_5* at both temperatures tested. The remaining combinations of *me_9* with DNA oligomers Nos. 213 and 207 did not result in any enhancement of RNA degradation, as expected (Fig 4).

### Analysis of antisense oligomer binding by thermodynamic calculations

A thermodynamic analysis of binding of the selected oligomers to the 5’UTR of CV-B3 was performed in order to explain some observations from the RNase H assay. Domains II, III and a part of domain IV were subjected to structure folding and the ability to bind antisense oligomers was analyzed by the *oligowalk* application in the *RNAstructure* program [27]. Binding of three DNA 19-mers and three RNA 16-mers was analyzed: DNA_162, DNA_213, DNA_420 as well as RNA5 (the oligomer corresponds to me_5), RNA8 (*me_8*) and RNA9 (*me_9*). The low binding energies exhibited by RNA_8 (-20.7 kcal/mol), RNA_5 (-17.9 or-18.2 kcal/mol depending on the structure variant) and RNA_9 (-15.8 kcal/mol) were consistent with their great binding abilities to the RNA target and made its structure more accessible to hybridization of DNA 19-mers (Table 2, Fig 3). The binding affinities of DNA 19-mers were lower and more variable. For DNA_162, the energy ranged between-8.2 kcal/mol for shorter domain II, and +4.5 kcal/mol for the extended version of this domain (Fig 2). DNA_213 exhibited binding energy of-8.9 kcal/mol to the pre-folded domain III, and DNA_420 was bound to its target with energy of -12.9 or-10.4 kcal/mol (when some internal base pairing was present in the loop). The results suggest that oligomer DNA_420 shows the best RNA binding abilities. However, in the presence of DNA_420, only weak induction of the RNase H activity was observed. It has been noted that for strong RNase H induction, a single-stranded 3’ end of the RNA-target-site before RNA-DNA duplex formation is important [7]. In the case of DNA_420, the 3’ end of the target site is predicted to be base paired (Figs 2 and 3). This might explain poor RNase H activation. In the case of DNA_162, the thermodynamic data suggest that only the shorter version of domain II would allow hybridization of an antisense oligomer.

**Table 2:** Binding energies (ΔG) of DNA 19-mers and RNA 16-mers to the target sites in the 5’UTR of CV-B3.

Oligomer name	Binding energy tosingle-stranded RNA [kcal/mol]	Binding energy to pre-folded RNA [kcal/mol]	Break target energy [kcal/mol]
**RNA_5**	-27.4	-17.9 (longer domain II)	-7.9
		-18.2 (shorter domain II)	-7.6
**RNA_8**	-27.7	-20.7	-7
**RNA_9**	-28.3	-15.8	-12.5
**DNA_162**	-18.6	+4.5 (longer domain II)	-23.1
		-8.2 (shorter domain II)	-10.4
**DNA_213**	-23.6	-8.9	-14.7
**DNA_420**	-19.9	-12.9	-7
		-10.4 (some base-pairing in the internal loop of domain IV)	-9.5

### Probing the secondary structure of targeted RNA *in vivo* with DMS

**Fig 5:**
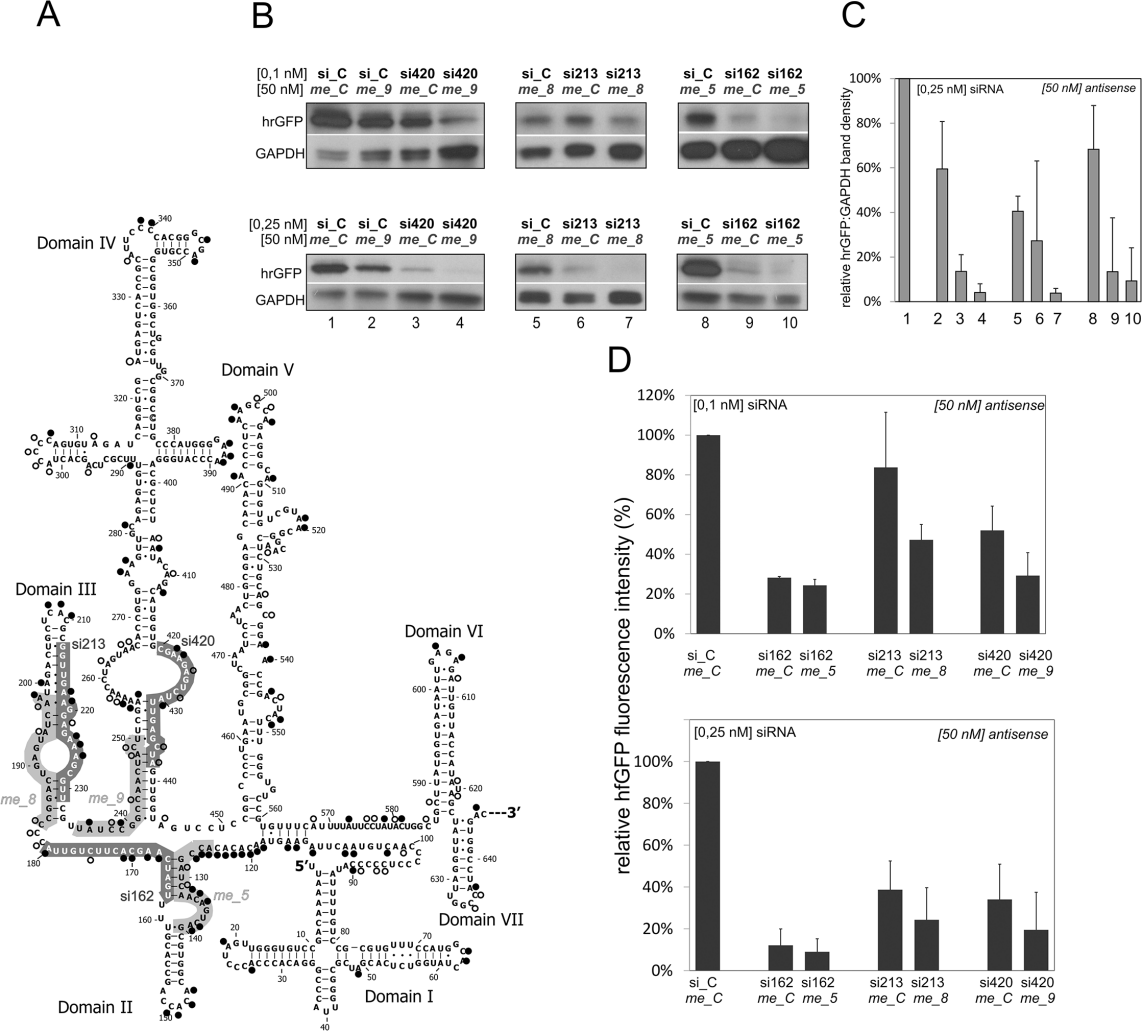
siRNA-induced silencing of the reporter construct hrGFP-5’UTRcvb3 in the absence and presence of helper oligomers. (A) Secondary structure model of the 5' UTR of CV-B3 according to Bailey and Tapprich (2007). Results of the *in vivo* structure probing are displayed on the model with the circles: black circle—strong DMS-induced A or C modification; empty circle—weak A or C modification. Thick gray lines indicate the target sites of helper 2’-O-methyl-16-mers (light gray) and siRNAs (dark gray). (B) Representative Western blot showing down regulation of the hrGFP-5’UTRcvb3 construct in HeLa cells. The cells were co-transfected with the combinations of 50 nM helper oligomers (name in italics) and 0.1 or 0.25 nM siRNA, respectively (name in black.: si162—siRNA_162, si213—siRNA_213, si420—siRNA_420), as indicated in the figure; GAPDH—loading control; hrGFP—reporter protein. Numbers under blots respect bars showed in graph in panel C. (C) Graph showing relative hrGFP band density normalized to GAPDH. Data were obtained in Western blots for 50 nM helper oligomers and 0.25 nM siRNA. Values are the averages from three independent experiments. Numbers under X axis respect lines showed in Western blot in panel B. The p-values were calculated using Student’s t-test for the following samples: Lanes 3:4, p = 0.062; lanes 6:7, p = 0.209; lanes 9:10, p = 0.756. (D) Graphs showing relative fluorescence intensity exhibited by HeLa cells co-transfected with oligomer combinations and a reporter fusion construct hrGFP-5’UTRcvb3, measured by plate reader. Components of the oligonucleotide mixture indicated in the figure as described above. The p-values were calculated using Student’s t-test for the following samples: 0.1 nM si162+*me_C*: 0.1 nM si162+*me_5*, p = 0.152; 0.1 nM si213+*me_C*: 0.1 nM si213+*me_8*, p = 0.147; 0.1 nM si420+*me_C*: 0.1 nM si420+*me_9*, p = 0.128; 0.25 nM si162+*me_C*: 0.1 nM si162+*me_5*, p = 0.679; 0.25 nM si213+*me_C*: 0.1 nM si213+*me_8*, p = 0.379; 0.25 nM si420+*me_C*: 0.1 nM si420+*me_9*, p = 0.451.

In order to establish whether the *in vitro* characterized secondary structure of the 5’UTR of CV-B3 remains unchanged in the intracellular environment, an *in vivo* structure probe was performed. To this end, HeLa cells infected at an moi 1 of CV-B3 (Nancy) were harvested, suspended in PBS and treated with 0.3% DMS (see Materials and Methods for details). The DMS-modified positions are shown in Fig 5A on the secondary structure model proposed by Bailey and Tapprich [32]. The results of the *in vivo* modification are in line with this model, with the exception that the proposed long-range interactions are not supported. However, domain II seems to be folded in a shorter form (Fig 2B) but not in a longer one (Fig 2A), which is characteristic for an alternative model of this region without long-range interactions.

### Down-regulation of reporter protein by siRNAs and their combinations with helper oligomers in HeLa cells

In the most effective three combinations of DNA 19-mers with helper 2’-O’-methyl-16mers, the DNA oligomers were replaced with corresponding siRNAs, directed against the same target sites in the 5’UTR of CV-B3. The following pairs of oligo tools were tested in a hrGFP-reporter assay in HeLa cells: siRNA_420 with *me_9*, siRNA_213 with *me_8* and siRNA_162 with *me_5* (Table 1). HeLa cells are widely used in transfection studies since they are generally well transfected by small oligomers and plasmid vectors, though an transfection protocol optimization is sometimes needed. The viral 5’UTR was cloned downstream the stop codons of the hrGFP sequence. Expression of such a construct resulted in the fused hrGFP-5’UTR mRNA. The mRNA construct was co-transfected with the respective combinations of siRNAs and 2’-O’-methyl-16mers. Subsequently, the expression level of hrGFP was established by fluorescence intensity measurements (Fig 5D) and it was visualised by Western blotting (Fig 5B and 5C). As shown in Fig 5, each siRNA at the concentration of 0.1 or 0.25 nM was capable of down-regulating the expression of the fusion hrGFP-5’UTR mRNA that resulted in partial silencing of hrGFP expression. The combinations of each siRNA with the respective helper 2’-O’-methyl-16mer used at 50 nM concentrations markedly increased the silencing effect. Combinations of siRNA and the control oligomer (i.e. the unspecific 2’-O’-methyl-16mer, *me_C*) did not reveal such effects (Fig 5). When applying helper oligomers which are modified to improve stability, it might be beneficial to test as low concentration as possible and find those that might work without causing any toxicity effect in the cell. It appeared that the combination of siRNA_162 with *me_5* was the most effective in silencing the hrGFP expression level. Up to 85% down-regulation at 0.25 nM concentration of siRNA was detected. Of the three tested combinations of siRNA and helper oligomer, the most pronounced improvement of the hrGFP down-regulation was observed for the combination of *me_8* and siRNA_213. As far as the down-regulation obtained by respective siRNA and *me_C* is concerned, more than 40% improvement in down-regulation occured at the an siRNA concentration of 0.1 nM.

## Discussion

Conserved regions of the virus genome are favorable target sites for antiviral siRNAs to prevent viral escape. However, since conserved viral regions are frequently highly structured and poorly accessible to the siRNA approach [15, 17, 18, 34], targeting such regions remains a challenge. Here, we described a procedure aimed at targeting a highly structured RNA region with specific siRNAs and helper antisense oligomers. Previously, helper oligonucleotides were successful employed to improve ribozyme-mediated cleavage of structured target RNAs [35]. In recent years, silencing experiments have primarily been carried out with RNAi approaches due to the superior efficiency of siRNAs compared to ribozymes. We therefore reasoned that helper oligomers might also improve binding of siRNAs to sites involved in the formation of tight structures. The 5’UTR of CV-B3 was chosen as the RNA target and the experimental flow leading to improving the efficiency of siRNA-based down-regulation was described in detail.

First, we mapped sites accessible to hybridization of complementary oligonucleotides within the 5’UTR of coxsackievirus B3. The secondary structure model for this RNA fragment has already been well-established [32]. However, it must be noted that accessibility to oligonucleotide hybridization does not reflect the RNA secondary structure in a simple, straightforward manner. Sometimes the partly double-stranded regions are more accessible to hybridization than the single-stranded, but AU-rich, stretches [23]. We expected that highly ordered structural elements present in the 5’UTR could also be an obstacle for oligomer binding. Therefore, two methods of accessibility mapping were used which are based on the random libraries of short DNA oligomers. We identified eight RNA stretches accessible to hybridization within the 741-nucleotide-long RNA target (Figs 1 and 2). Interestingly, only the 5’ sides of basal stems of domains II-VI turned out to be accessible. Domains VI, VII and large parts of domains I, IV and V were poorly accessible to oligomer hybridization. Importantly, the accessibility map did not cover all the single stranded regions in the secondary structure model of the RNA. Moreover, several proposed double-stranded stretches appeared to be accessible (Fig 2). A similar situation has previously been described for mapping experiments of other well-characterized RNA elements, of HDV and HCV [23, 24].

Short-oligomer libraries are very useful tools for mapping of sites accessible to oligonucleotide hybridization. However, an oligonucleotide of a slightly different length or complementary to sites shifted by only 1–2 nucleotides could result in significantly different RNA-binding characteristics. Therefore, the sites mapped as accessible with short-oligomer libraries were subsequently checked for their accessibility to longer DNA 16-mers using an RNase H assay. Not all regions found to be accessible to the shorter oligomers were able to bind longer oligomers. The first identified, efficient siRNA against 5’UTR of CV-B3[10], targets a linker between domain V and VI and a 5’-fragment of domain VI (nucleotide positions in the 5’UTR from 573 to 591). This target site was also mapped as accessible to hybridization in this study. Four out of seven stretches mapped as accessible by short-oligomer libraries also turned out to be accessible for DNA 16-mers and RNase H digestion, Nos. 1, 2, and 4–9 (Fig 3). Finally, domains V, VI were poorly accessible to hybridization of DNA 16-mers.

Interestingly, several regions accessible to hybridization consisted of a single-stranded stretch at their 5’ end, and a double-stranded segment at their 3’ ends. Such regions are not the best choice to attack since a free target 3’ end has been shown to be important for siRNA binding and efficacy [7]. In our search for the best potential target sites for the siRNA approach within the 5’UTR of CV-B3, 16 promising sites (siRNA-sites) were selected based on the RNA target sequence and siRNA design criteria. However, most of these sites were located outside the regions mapped as accessible or overlapped with them only partially (Fig 2). This was expected for such a structurally ordered and poorly accessible RNA region. There were some siRNA-target-sites identified within the regions mapped as accessible, which have been previously tested with DNA19-mers (siRNA-sites started with the nucleotide positions 238 and 583) or even with siRNAs (not shown), and found inactive or weakly active against CV-B3 [10].

An interesting observation was that several predicted siRNA-sites were located on the sides of the double-stranded stems opposite to the sites mapped as accessible. We decided to take advantage of this fact applying the approach of directed, local RNA structure change induced by helper oligomer hybridization, the ODMiR effect (*Oligonucleotide-Directed Misfolding of RNA*) [36]. To establish whether hybridization of helper oligomers results in inducing accessible sites on the opposite sides of the targeted domains, RNase H assays were performed. Four DNA 19-mers complementary to the selected siRNA-sites were tested either alone or in combination with fully-modified 2’-O-methyl-16-mers as helper oligomers. Each combination of a helper oligomer and siRNA was carefully chosen to avoid strong base-pairing between the two oligonucleotides. Thus, the target sites for both oligonucleotides could not be located exactly opposite one another on both sides of the double-stranded stem.

Three pairs of oligomers were found to be efficient in inducing RNase-H directed RNA cleavage: DNA_162 with *me_5*, DNA_213 with *me_8*, and DNA_420 with *me_9* (Fig 4). In these instances, simultaneous hybridization of the methylated helper 16-mer to the RNA resulted in increased RNase H cleavage on the opposite side of the stem, where the DNA 19-mer binds. Thus, hybridization of helper oligomers to the RNA made the second target site on the opposite side of a stem more accessible to binding of DNA 19-mer. The combinations of me_9 with DNA_213 or DNA_207 did not result in any enhancement of the RNA degradation level, which was expected, taking into account the secondary structure of this region.

The three target sites of the successful DNA 19-mers mentioned above were then used as siRNA target sites in cellular assays. Cell culture assays were carried out with a reporter construct enabling intracellular transcription of a fusion mRNA composed of the coding sequence of hrGFP and the 5’UTR of the CV-B3. Surprisingly, all three specific siRNAs applied separately were capable of down-regulating the expression of the fusion construct already at a low concentration of 0.1–0.25 nM (Fig 5). The siRNA_162 substantially reduced the reporter construct expression, although its DNA 19-mer counterpart (DNA_162) was only moderately active in the RNase H assay.

Finally, the three combinations of siRNA and helper oligomers were tested in cell culture. In a reporter assay we used a construct with hrGFP, although another reporter i.e. a dual-luciferase system could be also applied and it might be even more useful for quantitative analysis. The best improvement of hrGFP down-regulation was obtained for the combination of *me_8* and siRNA_213, at a concentration of 0.1 nM. Possibly, this was due to the relatively weak silencing effect of siRNA_213 alone. A significant improvement of the silencing effect was also observed for siRNA_420 in combination with helper oligomer *me_9*, despite the fact that siRNA_420 was targeted to the region previously mapped as inaccessible to hybridization. The best silencing potential against the 5’UTR-hrGFP was exhibited by siRNA_162 in a combination with 2’-O-methyl-16-mer *me_5*. At 0.25 nM concentration of these oligomers, expression of the reporter construct was inhibited by more than 90% (Fig 5). In the reporter assay, siRNA_162 was also very effective alone, although its DNA-19mer counterpart was only moderately able to bind domain II *in vitro*. That observation corresponds to our results of the *in vivo* structure probing: the less structured form of domain II existing *in vivo* could explain its accessibility to the siRNA approach. This suggestion is in line with some recent findings showing that RNA molecules *in vivo* seem to remain more denatured in comparison with the *in vitro* conditions [37].

In the present work, we demonstrated successful targeting of a highly structured RNA with siRNAs, that were used in combination with helper oligomers, 2’-O-methyl-16mers, in living human cells. While many mRNAs can efficiently be silenced by means of RNAi, some stuctured RNAs have proven refractory to siRNA binding. Our experimental procedure might facilitate the proper design of nucleic acid-based tools to target highly structured RNA targets, including the 5’UTRs of pathogenic RNA viruses.

## Supporting Information

S1 Fig Mapping of sites accessible for oligonucleotide hybridization in 5’UTRcvb3 RNA with the RT-ROL method. (TIF)

S2 Fig Analysis of the RNA secondary structure of the 5’UTR of CV-B3 with DMS *in vivo*. (TIF)

S1 Table DNA primers which were used in PCR reaction amplifying the RT-ROL products. (PDF)

S2 Table Antisense DNA oligomers which were used in the RNase H assay. (PDF)

S3 Table DNA primers which were used in reverse transcription reaction following RNA structure probing *in vivo*. (PDF)
