# Supplementary figures and images for: Targeting Highly Structured RNA by Cooperative Action of siRNAs and Helper Antisense Oligomers in Living Cells
Source: PLoS One. 2015 Aug 26;10(8):e0136395. doi: 10.1371/journal.pone.0136395 (PMC4556297; doi:10.1371/journal.pone.0136395)

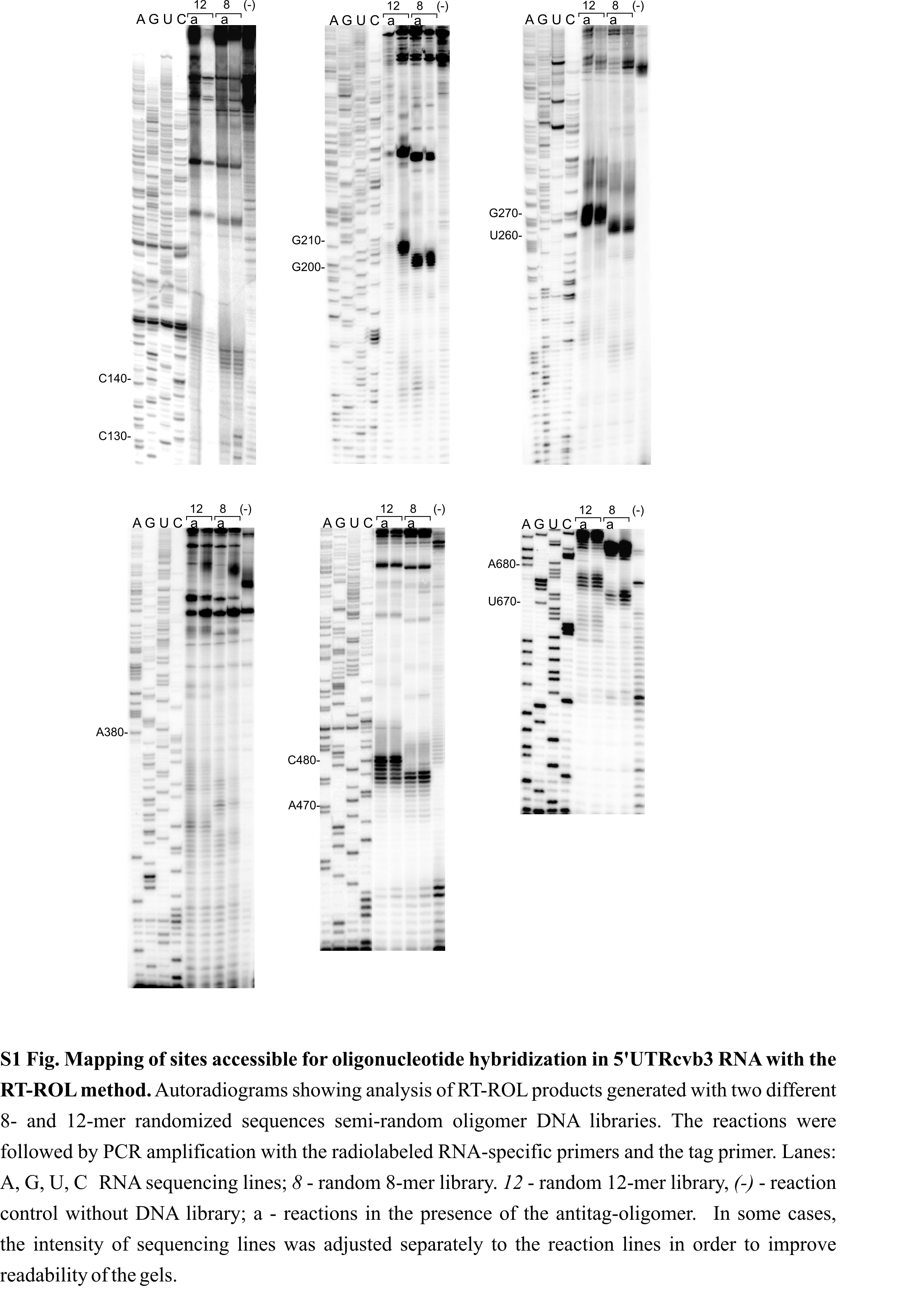

Supplement: S1 Fig — (TIF) [file pone.0136395.s001.tif]

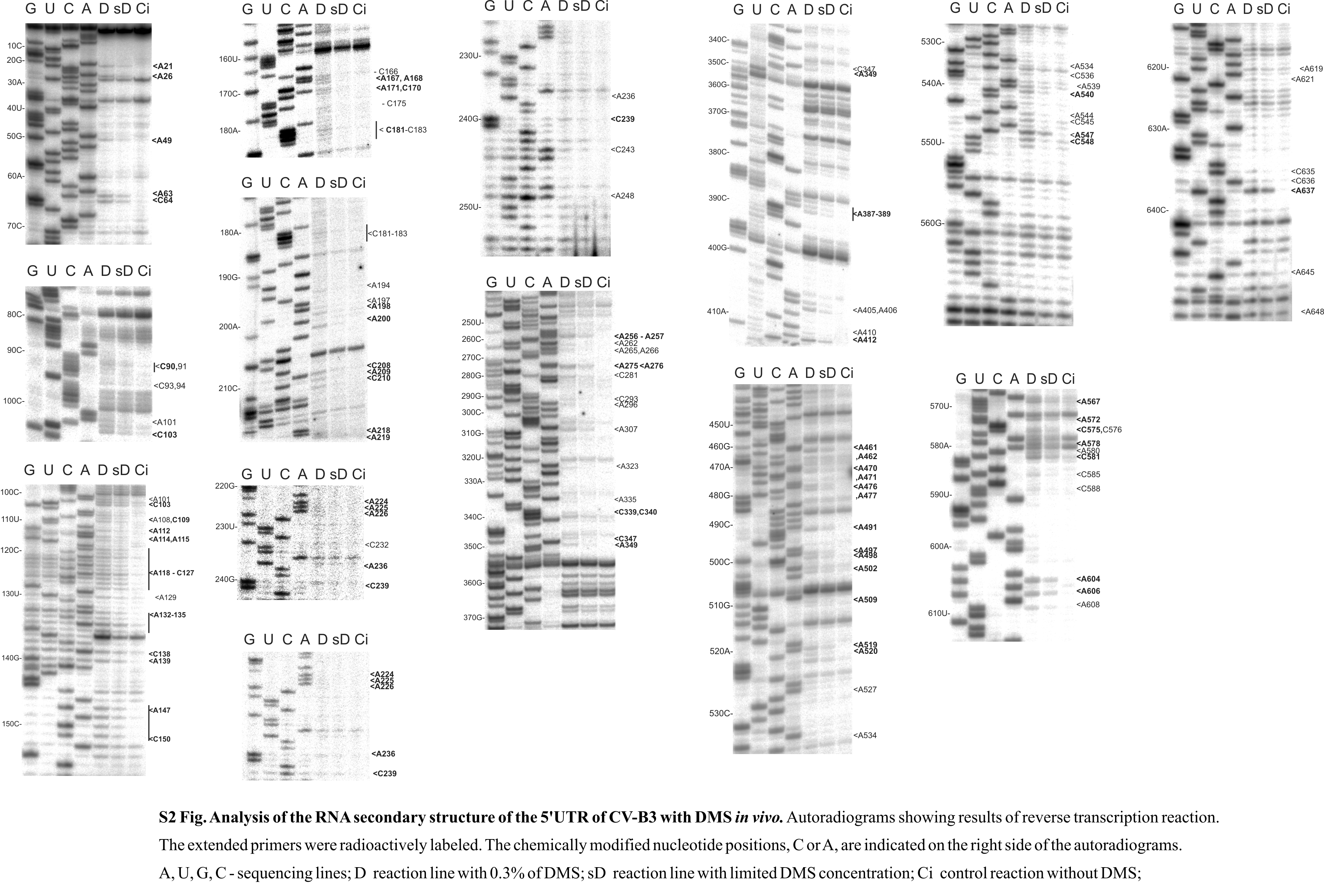

Supplement: S2 Fig — (TIF) [file pone.0136395.s002.tif]
