# Supplementary material for: Targeting Highly Structured RNA by Cooperative Action of siRNAs and Helper Antisense Oligomers in Living Cells
Source: PLoS One. 2015 Aug 26;10(8):e0136395. doi: 10.1371/journal.pone.0136395 (PMC4556297; doi:10.1371/journal.pone.0136395)
Supplement: S1 Table — (PDF) [file pone.0136395.s003.pdf]

**S1 Table. DNA primers which were used in PCR reaction amplifying the RT-ROL products.**

| **Name of DNA oligomer** | **sequence 5'-3'** |
|---|---|
| TAG16 | CTTAAGGTAGGACTAC |
| St 93-113 | CTCCCCCAACTGTAACTTAG |
| St 133-151 | CAGTCAGCGTGGCACACC |
| St 182-205 | CCGGACTGAGTATCAATAGACTG |
| St 308-330 | TGTAGATCAGGTCGATGAGTCA |
| St 430-451 | TTGAGCTAGTTGGTAGTCCTC |
| St 548-567 | CTTTGGGTGTCCGTGTTTC |
| St 626-642 | TTGGATTGGCCATCCG |
