# Supplementary material for: Targeting Highly Structured RNA by Cooperative Action of siRNAs and Helper Antisense Oligomers in Living Cells
Source: PLoS One. 2015 Aug 26;10(8):e0136395. doi: 10.1371/journal.pone.0136395 (PMC4556297; doi:10.1371/journal.pone.0136395)
Supplement: S2 Table — (PDF) [file pone.0136395.s004.pdf]

S2 Table. Antisense DNA oligomers which were used in the RNase H assay.

Name of DNA 16-mers	sequence 5'-3'	complementary region in 5'UTRcvb3
**1**	TACAGTTGGGGGAGGG	92-107
**2**	GTTGATCGGTGTGTGT	119-134
**3**	ACGCTGACTGTTGATC	128-143
**4**	ACTGTTGATCGGTGTG	122-137
**5**	CTGACTGTTGATCGGT	125-140
**6**	CTCAGTCCGGGGTAAC	177-192
**7**	TACTCAGTCCGGGGTA	179-194
**8**	TTGATACTCAGTCCGG	183-198
**9**	AGTAGTTGGCCGGATA	235-250
**10**	CGAAGTAGTTGGCCGG	238-253
**11**	TTTCGAAGTAGTTGGC	241-256
**12**	TTCAGGGGCCGGAGGA	447-462
**13**	GCATTCAGGGGCCGGA	450-465
**14**	CCATAAGCAGCCAGTA	579-594
**15**	TATAATAGCTCTATTA	647-662
**16**	GATATATAATAGCTCT	651-666
**17**	AGGGATATATAATAGC	654-669
**18**	CAAAGGGATATATAAT	657-672
**19**	CCAACAAAGGGATATA	661-676
**C**	TACGATAGCGCTAGCT	-
**Name of DNA 19-mers**		
**DNA_162**	TAACAGAAGTGCTTGATCA	162-181
**DNA_207**	TTCTCCTTCAACCGCGTGA	207-226
**DNA_213**	AACGCTTTCTCCTTCAACC	213-232
**DNA_420**	TAGCTCAATAGACTCTTCG	420-438
**DNA_C (19-mer)**	ACGTGACACGTTCGGAGAA	-
