# Supplementary material for: Targeting Highly Structured RNA by Cooperative Action of siRNAs and Helper Antisense Oligomers in Living Cells
Source: PLoS One. 2015 Aug 26;10(8):e0136395. doi: 10.1371/journal.pone.0136395 (PMC4556297; doi:10.1371/journal.pone.0136395)
Supplement: S3 Table — (PDF) [file pone.0136395.s005.pdf]

S3 Table. DNA primers which were used in reverse transcription reaction following RNA structure probing *in vivo*

**Name of DNA oligomer**	**sequence 5'-3'**
ST 99-117	ACTTCTAAGTTACAGTTGG
ST 163-181	GTAGCAGAAGTGCTTGATC
ST 260-278	ACTTCCACGGTGTTACTAG
ST 358-376	AGGCCGCCAACGCAGCCAC
ST 457-475	AGGATTAGCCGCATTCAGG
ST 573-591	TAAGCAGCCAGTATAGGAA
ST 653-670	AAGGGATATATAATAGCTC
